# Network pharmacology-guided optimization of Semen Sojae Praeparatum fermentation for enhanced anti-influenza efficacy

**DOI:** 10.1038/s41598-026-47737-7

**Published:** 2026-04-09

**Authors:** Wenping Liu, Wenlong Zhao, Qian Zhang, Xiaofei Wu, Tingting Hu, Yuejian Zhu

**Affiliations:** 1 College of Traditional Chinese Medicine, Bozhou University, No. 2266, Tangwang Avenue, High-tech Zone, Bozhou, 236800 Anhui Province China; 2 Chinese Medicine Department, Anhui Traditional Chinese Medicine Science and Technology School, No.1 Lianhua Road, Economic Development Zone, Bozhou, 236800 Anhui Province China; 3 Anhui Jiren Pharmaceutical Group Traditional Chinese Medicine Research Institute Co Ltd, No. 11, Zone B, Chinese Herbal Medicine Product Entrepreneurship Base, Bozhou Economic Development Zone, Bozhou, 236800 Anhui Province China

**Keywords:** Sojae Semen Praeparatum, Influenza, Fermentation optimization, Fingerprint, Biochemistry, Computational biology and bioinformatics, Drug discovery

## Abstract

Semen Sojae Praeparatum (SSP), a traditional Chinese medicine derived from fermented black soybeans, has been historically used to treat febrile epidemics (“Wen Bing”) with symptoms akin to influenza. This study integrated network pharmacology, molecular docking, in vitro pharmacodynamics and HPLC-based fingerprint and quality evaluation to elucidate SSP’s anti-influenza mechanisms and optimize its fermentation process. Network pharmacology identified genistein and daidzein as key bioactive constituents targeting hub proteins TNF, AKT1, EGFR, SRC, and MMP9, which mediate pathological process in influenza. Molecular docking confirmed their strong binding affinities, particularly to lung-expressed AKT1 and SRC both of which are upstream activating kinases of the NF-κB p65 signaling pathway. Guided by these computational predictions, genistein and daidzein were evaluated in A549 cells using LPS and H_2_O_2_-induced influenza lung injury models. They significantly inhibited NF-κB p65 nuclear translocation, suppressed transcription and secretion of pro-inflammatory cytokines (TNF-α, IL-1β, IL-8, PTGS2), upregulated antioxidant defense genes (NQO1, GCLM, SOD2), and reduced oxidative stress markers (ROS, MDA), thereby highlighting SSP’s potential protective effects during influenza-induced pulmonary infection. Concurrently, SSP fermentation parameters were optimized (water-to-material ratio of 20:1 (v/w), 30 ± 2 °C, open conditions), yielding nine SSP batches with high chromatographic similarity (> 0.989) and genistein/daidzein total contents exceeding Chinese Pharmacopoeia standards. Compared to commercial products, optimized SSP showed significantly higher indicator constituents’ levels and superior quality consistency. This study establishes a robust, scalable framework for standardized SSP production, enhancing its clinical reliability and industrial applicability in influenza management.

**Supplementary Information:**

The online version contains supplementary material available at 10.1038/s41598-026-47737-7.

## Introduction

Influenza, commonly known as the flu, remains one of the most pervasive and burdensome infectious diseases worldwide, posing significant threats to public health. Each year, seasonal epidemics affect an estimated 5–10% of adults and 20–30% of children globally, resulting in approximately 1 billion cases, 3–5 million severe illnesses^1^, and over 290–650 thousand respiratory deaths^2^. Beyond acute morbidity and mortality^3^, influenza exacts a profound socioeconomic toll through lost productivity, healthcare system strain, and vulnerability to secondary complications, particularly in high-risk populations such as the elderly, young children, and those with comorbidities^4^. In influenza management, Traditional Chinese medicine (TCM) offers distinct advantages. By leveraging multi-component, multi-target mechanisms, TCM addresses viral replication, excessive inflammation, and immune dysregulation holisticall^5,6^. They have demonstrated superior symptom relief, fever reduction, and shorter recovery times compared to using antivirals like oseltamivir alone^7^. These benefits stem from TCM’s ability to modulate cytokine storms, inhibit pro-inflammatory pathways, and bolster host defenses, providing broader coverage against variant strains and reducing reliance on single-target drugs^8,9^.

Among TCM agents, Semen Sojae Praeparatum (SSP, Dan Dou Chi, fermented black soybean) derived from the mature seeds of *Glycine max* (L.) Merr. through controlled fermentation with adjuncts like Sweet Wormwood Herb and Mulberry Leaf^10^. It rich in bioactive constituents, including isoflavones, polysaccharides, saponins, proteins, and oligosaccharides^11–13^. Due to these constituents, SSP exhibits multifaceted pharmacological actions: pungent, bitter, and cool, entering the lung and stomach meridians to dispel exterior pathogens, clear heat, resolve toxins, and alleviate irritability^14^. Historically, its application traces to ancient texts like “Shennong herbal scripture”, where it was prescribed for febrile epidemics (“Wen Bing”) and wind-cool invasions manifesting as chills, fever, headache, and restlessness hallmarks of influenza-like illness^15,16^.

Numerous TCM formulations incorporating SSP have been extensively employed for the management of influenza^17,18^, leveraging its properties to dispel exterior pathogens. Accordingly, SSP’s quality significantly impacts its efficacy in treating influenza-related external diseases. However, SSP’s production of faces challenges related to inconsistent quality, primarily attributable to variability in fermentation environments, microbial consortia, and processing parameters^19^, which influence bioactive constituent profiles and lead to heterogeneous therapeutic efficacy. To address these limitations, this study employs a network pharmacology approach to identify the bioactive constituents in SSP responsible for its anti-influenza effects. Leveraging these findings, in vitro anti-influenza effects of identified bioactive constituents evaluated in A549 human lung epithelial cells. Building upon these results, SSP fermentation protocols were optimized by systematically evaluating key parameters, including the water-to-material ratio, fermentation temperature, and sealing efficacy. Subsequently, HPLC fingerprinting was performed to assess the chemical consistency of the fermented products. This integrated methodology provides robust experimental evidence to support standardized SSP production, thereby enhancing its reliability and clinical applicability for influenza therapy.

## Materials and methods

### Network pharmacology analysis

#### SSP’s bioactive constituents screening and influenza-associated targets identification

SSP-related constituents were extracted from the HERB database (http://herb.ac.cn/) constituents and screened using Lipinski’s Rule of Five, prioritizing molecular weight ≤ 500 Da, hydrogen bond donors ≤ 5, hydrogen bond acceptors ≤ 10, and lipid-water partition coefficient (LogP) ≤ 5. SMILES of selected constituents were analyzed in SwissTargetPrediction (http://swisstargetprediction.ch/) to identify gene targets with interaction probabilities > 0.5. Influenza-related genes were retrieved from GeneCards (https://genecards.weizmann.ac.il/v3/) using the keyword “influenza,” with targets filtered by a Relevance Score ≥ 0.7. The SSP active constituents and influenza gene targets were analyzed using Venny (https://jvenn.toulouse.inra.fr/) to identify common targets via intersection analysis, visualized in a Venn diagram to reveal potential therapeutic mechanisms.

#### PPI network construction and functional enrichment analysis

The predicted constituent-target interaction pairs obtained from SwissTargetPrediction were organized into a two-column table (constituents and corresponding target) with one-to-one correspondence. This table was imported into Cytoscape (http://www.cytoscape.org/, Version 3.10), where the SSP-active constituent-intersection target network was constructed and visualized. Network topology analysis was subsequently performed using Cytoscape’s built-in “Analyze Network” function to calculate the overall network structure as well as topological parameters (SSP’s constituents and intersection target) for each node and edge.

The intersecting targets identified from Venny analysis were submitted to the STRING database (https://string-db.org/) to construct a protein-protein interaction (PPI) network excluding unconnected nodes (filtered with degree threshold ≥ 1). Concurrently, these targets were analyzed in the DAVID database (https://davidbioinformatics.nih.gov/ and https://www.kegg.jp/kegg/kegg1.html) for KEGG pathway^20–22^ and Gene Ontology (GO) enrichment analysis, with results visualized using GraphPad Prism (Version 9.0, GraphPad Software). The PPI network data were analyzed and visualized with Cytoscape.

#### Molecular docking validation

Key bioactive constituents shared between the SSP-active constituent-intersection target network and their association with the top hub targets identified from the PPI network of intersection targets, were selected for molecular docking. Three-dimensional structures of the constituents were obtained from PubChem in SDF format, converted to PDB format using Open Babel, and processed in PyRx (Version 0.8, https://pyrx.sourceforge.io/) by defining torsions and exporting as PDB files. Protein structures of the selected targets were retrieved from the Protein Data Bank (PDB), refined using Discovery Studio (Version 4.5, BIOVIA), and saved in PDB format before being converted to PDBQT files in PyRx. Docking was performed with AutoDock Vina (Version 2.0), integrated within PyRx.

### In vitro anti-inflammatory and antioxidant assays

#### Bioactive constituents’ concentration screening

Human lung adenocarcinoma A549 cells (National Biomedical Laboratory Cell Repository, China) were cultured in DMEM medium (Zhongke Maichen (Beijing) Technology Co., Ltd.) supplemented with 10% fetal bovine serum, 100 U/mL penicillin, and 100 µg/mL streptomycin (Hyclone, Thermofisher, China). Cells were maintained at 37 °C in a humidified atmosphere with 5% CO₂ incubator (Thermo Fisher Scientific, USA). Genistein and daidzein (Nanjing Yuanzhi Biotechnology Co., Ltd., China) were dissolved in DMSO to prepare 300 mM stock solutions Stock solutions were serially diluted 2-fold to obtain a concentration gradient spanning 300 mM down to 0.5859 µM for the preliminary cytotoxicity screening.

A549 cells were seeded in 96-well plates at 5 × 10³ cells/well and incubated for 24 h. Cells were treated with serial dilutions of genistein or daidzein for 2 h pretreatment and with LPS (1 µg/mL) or H_2_O_2_ (400 µM) for another 24 h. Cell viability was assessed using MTT assay (Beyotime Biotechnology, China), reactive oxygen species (ROS) levels were quantified using the DCFH-DA probe (Beyotime Biotechnology, China), nitric oxide (NO) levels were determined using a Griess reagent kit (Beyotime Biotechnology). EC50 values were calculated for further experiments with GraphPad Prism 9.0 (GraphPad Software, USA).

#### Quantitative real-time PCR and western blot analysis

Following treatments, total RNA was extracted using TRIzol reagent (Invitrogen, Carlsbad, CA, USA). cDNA was synthesized with All-In-One RT MasterMix (Abmgood, China). qPCR was performed on 7500 Real-Time PCR System (Applied Biosystems, USA) using SYBR Green Master Mixes (KAPA Biosystems, South Africa). Primers for target genes were listed in Table S1. Relative expression was calculated using the 2^-ΔΔCt method.

Nuclear and cytoplasmic proteins were extracted using nucleus/cytoplasmic protein extraction using nucleus protein extraction kit (Solarbio, China). Protein concentrations were determined by BCA assay (Beyotime Biotechnology, China). Equal amounts (20 µg) were separated by 10% SDS-PAGE and transferred to PVDF membranes (Millipore, Billerica, MA, USA). Membranes were blocked with 5% non-fat milk in TBST for 1 h, incubated with primary antibodies (anti-p65 NF-κB, anti-GAPDH and anti-H3; Servicebio, China; 1:1000 dilution) overnight at 4 °C, and then with HRP-conjugated secondary antibodies (1:5000) for 1 h. Bands were visualized using ECL reagent ((Biosharp, China) and quantified with ImageJ software (NIH, Bethesda, MD, USA).

#### Inflammatory cytokines and antioxidant factors detection

Supernatants from treated cells were collected and centrifuged at 1000 × g for 10 min. TNF-α and IL-1β levels were measured using commercial ELISA kits (Nanjing Jiancheng Bioengineering Institute, China). ROS and malondialdehyde (MDA) content was measured using commercialized assay kit (Nanjing Jiancheng Bioengineering Institute, China), following manufacturers’ instructions. Absorbance was read using SpectraMax M5 multimode reader (Molecular Devices, China).

All experiments were performed in triplicate, and data were analyzed using GraphPad Prism 9.0 (GraphPad Software, USA). Statistical significance was determined by one-way ANOVA followed by Tukey’s post-hoc test (*p* < 0.05).

### Quality evaluation of SSP

#### Optimization of SSP fermentation process

**Fig. 1:**
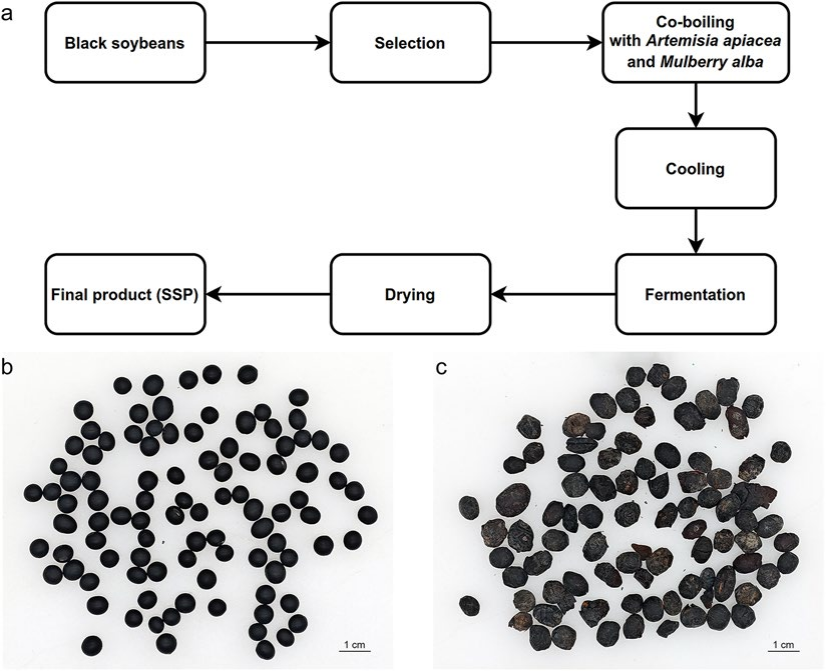
Fermentation process and production of SSP. (**a**) was the fermentation process, (**b**) was black soybeans, the ingredient of SSP fermentation, (**c**) was the SSP fermented products.

The preparation process of SSP is depicted as follows: Sweet Wormwood Herb and Mulberry Leaf (as called auxiliary materials, Bozhou Huqiao Pharmaceutical Co., Ltd.), each at 10% of the net weight of black soybeans (Shandong Shouliang Food Technology Co., Ltd.), were combined with water and boiled together for 3 h until thoroughly cooked. The water volume was adjusted based on the total material input. The herbal leaves were enclosed in a medicinal gauze bag or cloth during boiling. After cooking, the leaves were removed, and the soybeans were cooled and transferred to either a fermentation chamber or a controlled fermentation box (WL-6F1, Zhongshan We Lai Electrical Appliance Technology Co., Ltd.). The material was spread evenly for fermentation. In the fermentation box, incubation was maintained at a constant temperature for 10–15 days until a yellow coating (indicative of successful fermentation) appeared. Alternatively, in the fermentation chamber, the material was covered with clean, airtight plastic film on all sides and fermented at ambient temperature for 10–15 days. The fermented product was then removed, washed, and dried in a forced-air constant-temperature drying oven (Model DGT-G70, Huadeli Scientific Equipment Co., Ltd.) at 40 °C to obtain the final SSP (Fig. 1c).

Three single-factors of fermentation processes were evaluated: water-to-material ratio, fermentation temperature and sealing conditions, the arrangement and combination listed in Table 1.

**Table 1:** Fermentation condition.

Batch	Water-to-material ratio	Temperature (℃)	Sealing condition
1	16:1	30 ± 2	Open
2	16:1	30 ± 2	Sealed
3	18:1	30 ± 2	Open
4	18:1	30 ± 2	Sealed
5	20:1	30 ± 2	Open
6	20:1	30 ± 2	Sealed
7	16:1	25 ± 2	Sealed
8	18:1	25 ± 2	Sealed
9	20:1	25 ± 2	Sealed

#### Fingerprint analysis and content determination

HPLC was conducted using an LC-16 system (Shimadzu Instrument Co., Ltd., Suzhou, China) equipped with a C18 reverse-phase column (4.6 mm × 250 mm × 5 μm, Suyuan Medical Technology Co., Ltd., Suzhou, China). The mobile phase consisted of a 25:75 (v/v) mixture of acetonitrile and water containing 1% acetic acid, delivered at a flow rate of 1.0 mL/min under isocratic conditions at 30 °C for a total runtime of 85 min. An injection volume of 10 µL was utilized, with analyte detection performed at a wavelength of 260 nm.

Network pharmacology analysis selected anti-influenza, constituents: genistein and daidzein, (Chengdu Alfa Biotechnology Co.,Ltd.) were accurately weighed and diluted with methanol to concentrations of 200 µg/mL as standard solutions.

The fingerprint analysis was conducted by importing the chromatograms of 9 batches of SSP samples Similarity Evaluation System for Chromatographic Fingerprint of Traditional Chinese Medicine (SESCFTCM, Version 2012, Beijing, Chinese Pharmacopoeia commission) of the for analysis. The control chromatogram (S1) was set as the average value method, with the time window width set at 0.5.

#### Routine quality assessment

Impurity assessment: impurities in SSP primarily consist of inorganic contaminants such as sand, mud, dust, residual auxiliary materials, and deteriorated or moldy products. They were weighed and the percentage content in the sample was calculated. The total impurity content in SSP must not exceed 3.0%.

Moisture determination: Samples (3 g) was ground into particles ≤ 3 mm, placed in a pre-weighed flat weighing bottle (spread ≤ 5 mm thick, loose material ≤ 10 mm), and accurately weighed. The bottle was uncovered and dried at 100–105 °C for 5 h, then covered, cooled in a desiccator for 30 min, and reweighed. The process was repeated after air-drying at ambient temperature for 1 h until the weight difference was < 5 mg. Moisture content (%) was calculated based on mass loss, with a maximum allowable limit of 12.0% for SSP.

Total ash content analysis: Samples was pulverized to pass through sieve of size 2 and mixed uniformly. Samples (2–3 g) was accurately weighed into a pre-ignited, constant-weight crucible. The sample was gradually heated to carbonize without ignition, then ashed at 500–600 °C until constant weight was achieved. Total ash content (%) was determined based on the residual mass, with a maximum limit of 8.0% for SSP.

Extractum determination: Samples were ground to pass through sieve of size 2 and homogenized and precisely weighed into a conical flask with 25 times water. After soaking for 1 h, the mixture was refluxing extraction for 1 h. The solution was filtered through a dry filter, and 25 mL of filtrate was transferred to a pre-dried, constant-weight evaporating dish and dried at 105 °C for 3 h. Water-soluble extractive content (%) was calculated on a dried basis, with a minimum requirement of 15.0% for SSP.

SSP samples (1 g) sieved with size 2 mesh were accurately weighed and transferred into a stoppered conical flask. Precisely 25 mL of methanol was added. The mixture was refluxed with heating for 1 h and the lost weight was compensated with methanol. Subsequently, the extracting solution was filtrated and collected for analysis.

## Results

### Candidate constituents and targets of anti-influenza

**Fig. 2:**
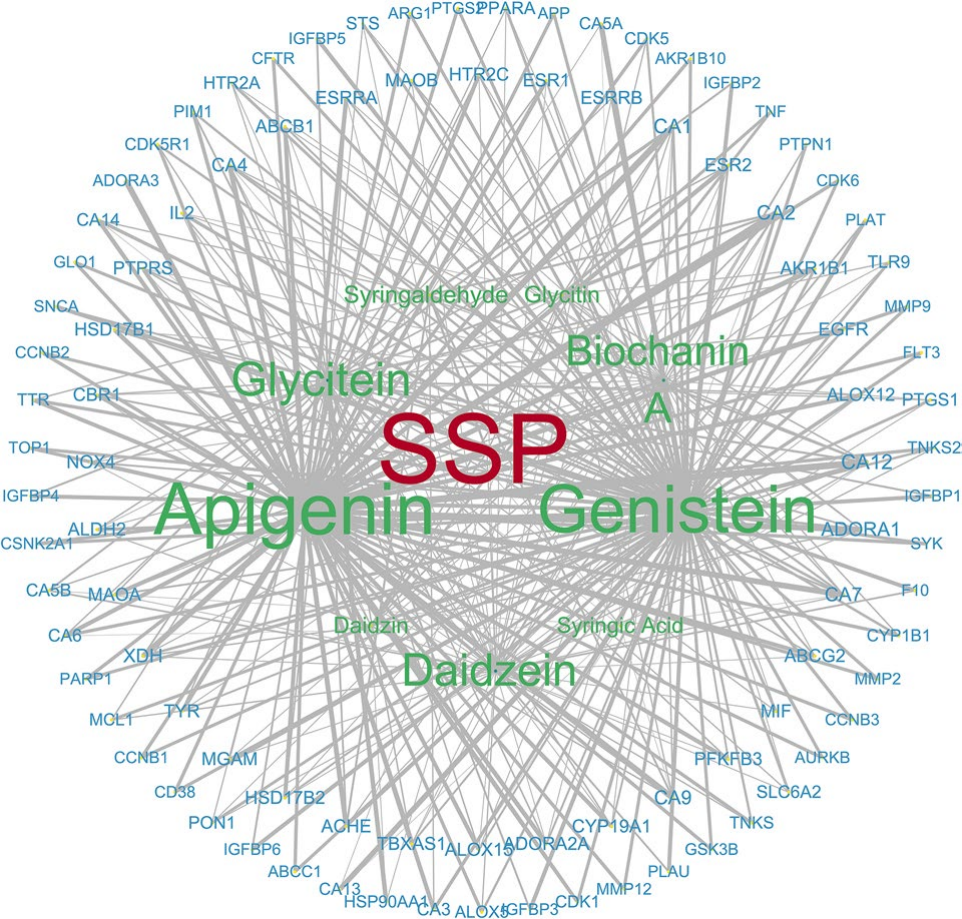
Network of intersecting targets for major constituents in SSP. The type of elements was classified with color: blue target gene, green constituents in SSP, red SSP. Node size and line width in the network visualization represented degree and edge-betweenness values respectively, with line width indicating greater connectivity in the local network.

**Fig. 3:**
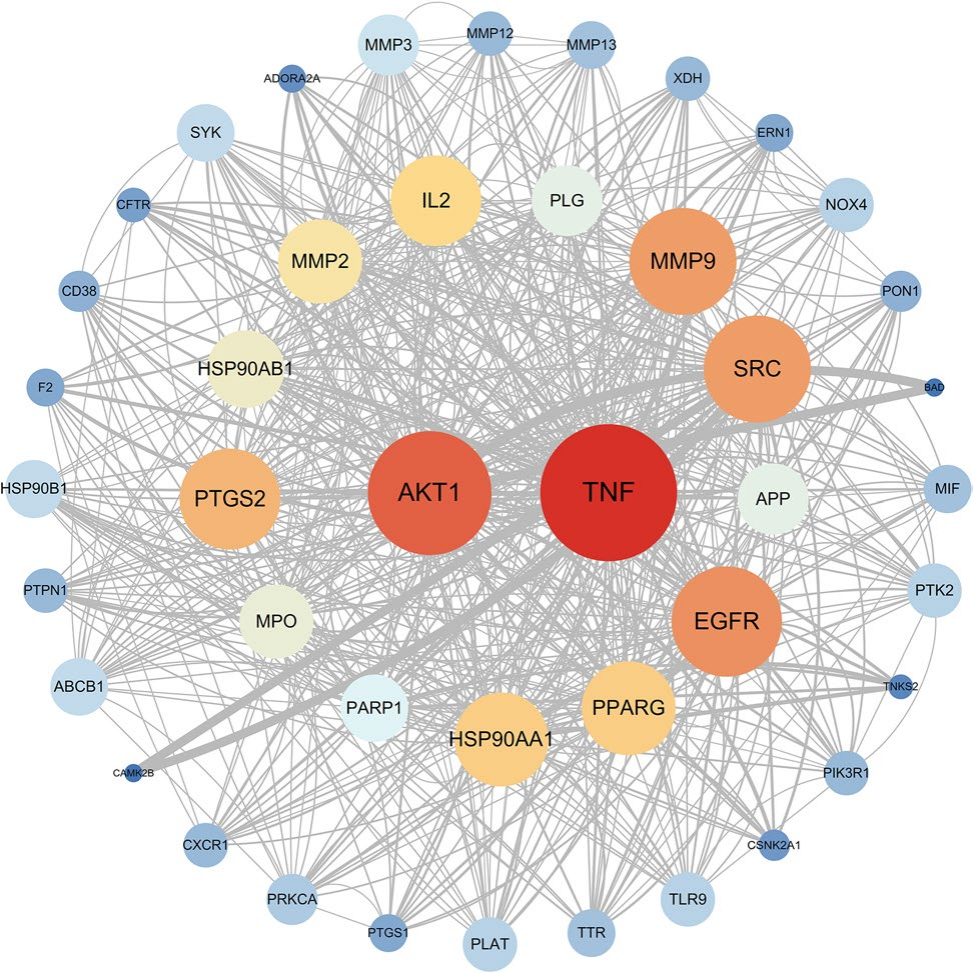
Topological analysis diagram of potential anti-influenza PPI targets of SSP. In the network visualization, node size and color represented degree values, while edge width indicated edge-betweenness centrality. Larger or redder nodes, as well as wider edges, indicated greater connectivity and functional significance within the local network.

Active constituents of SSP were extracted from the HERB database and filtered by Lipinski’s Rule of Five, yielding 11 candidates (Table S2). The number of overlap targets between SSP and influenza was 45 (Fig. S1). Five core constituents were selected by sorting in descending order of degree value, revealing genistein, apigenin, glycitein, daidzein, and biochanin A (Fig. 2). The overlap targets were subsequently processed in STRING to build a PPI network (Fig. S2), with degree values computed. The five highest-degree hub targets: TNF, AKT1, EGFR, SRC, and MMP9, emerged as pivotal mediators of influenza mediators (Fig. 3).

### KEGG and gene ontology (GO) enrichment analysis

**Fig. 4:**
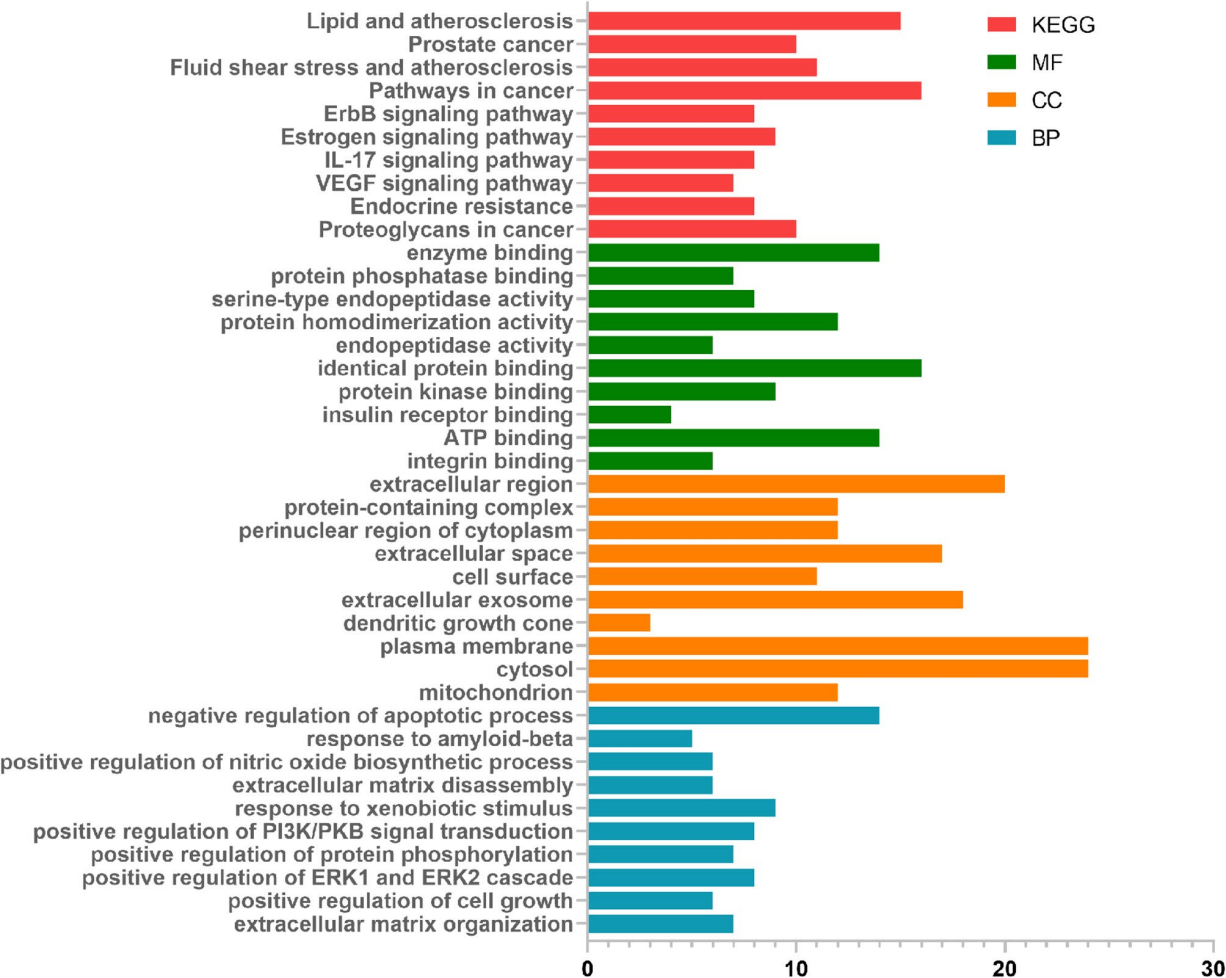
KEGG and GO enrichment analysis of SSP. The bar plot displayed the number of overlapping targets between SSP and influenza. Enrichment results were color-coded: red for KEGG pathways, green for molecular functions (MF), orange for cellular components (CC), and blue for biological processes (BP).

KEGG and GO enrichment analysis of the 109 intersection targets in DAVID identified 118 KEGG pathway, 78 molecular function (MF) terms, 47 cellular component (CC) and 241 biological processes (BP). The top enriched KEGG and GO terms, ranked by ascending *p*-value, were depicted as bar plots in Fig. 4. These enriched terms align with four major pathological domains of influenza: (1) Cytokine storm and hyperinflammation (via IL-17, VEGF, and nitric oxide pathways); (2) Viral replication and host cell survival (via PI3K/AKT, ErbB, and anti-apoptotic regulation); (3) Pulmonary tissue remodeling and injury (via MMP-driven ECM disassembly and integrin-mediated adhesion); and (4) Endothelial dysfunction and vascular leakage (via fluid shear stress, atherosclerosis, and VEGF signaling). Collectively, the initiation and progression of influenza were the result of the combined effects of multiple factors. Therefore, the anti-influenza mechanisms of SSP were multi-dimensional framework.

### Molecular docking validation of key anti-influenza constituents and targets

**Fig. 5:**
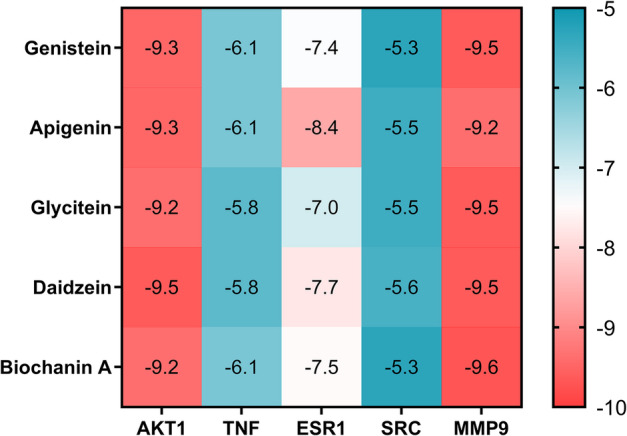
The binding energy between key constituents and targets. In the docking matrix, binding energies between constituents and target proteins were visualized using a color gradient: redder hues indicated stronger affinity (more negative energies), while bluer hues denoted weaker affinity (less negative energies).

**Fig. 6:**
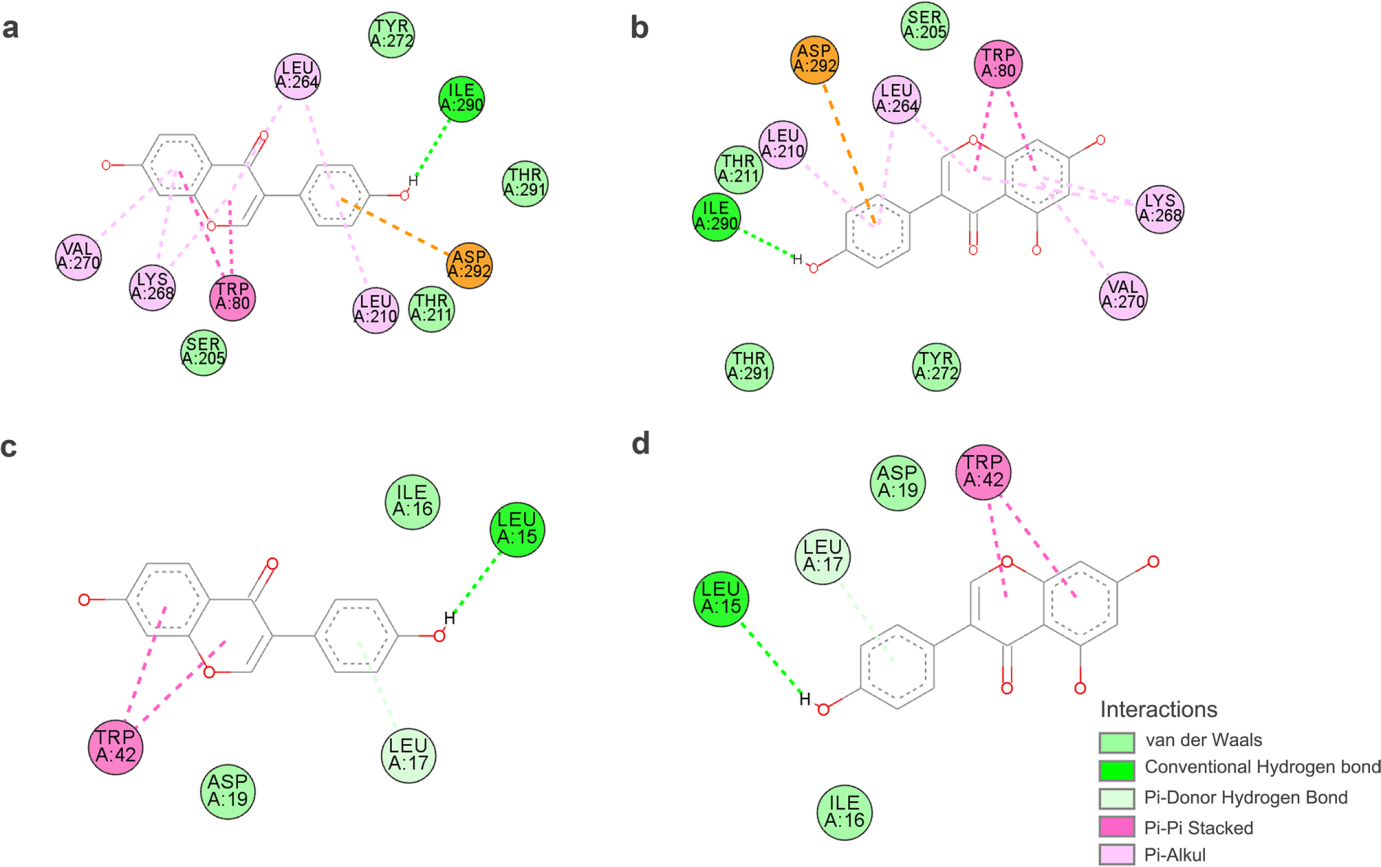
Molecular docking visualizations of key constituents-target interactions. (**a** and **b**) was the interactions of genistein and daidzein with AKT1, respectively; (**c** and **d**) was their interactions with SRC. Common interaction modes included van der Waals forces, Pi-Pi Stacked and Pi-Alkyl.

To investigate the interactions between key active constituents and critical targets, five constituents (genistein, apigenin, glycitein, daidzein, and biochanin A) were identified as overlapping key constituents from the SSP-constituent-target network. These constituents were docked against five hub targets TNF, AKT1, EGFR, SRC, and MMP9) selected from the protein-protein interaction (PPI) network based on their top degree values. Molecular docking was performed using the target proteins as receptors and the active constituents as ligands, yielding 25 docking pairs. Binding energies were analyzed and visualized as a heatmap (Fig. 5). All binding energies were negative, ranging from − 5 to -10 kcal/mol. These results indicate robust binding interactions between SSP’s key anti-influenza constituents and critical targets. Given that children and the elderly represented the primary susceptible populations for influenza and the lungs serve as the predominant site of infection, we selected AKT1 and SRC (both exhibiting high pulmonary expression, Table S3) as key evaluation metrics and calculated the weight binding energy (Table 2). These two targets are pivotal host factors exploited by influenza viruses to facilitate infection, replication, and inflammatory responses. Their dysregulation contributes to disease progression, including cytokine storm and lung injury in influenza, which align with the “Wen Bing” addressed by SSP. Genistein, daidzein and apigenin emerged as the top three constituents exhibiting the highest binding affinities to AKT1 and SRC. Owing to apigenin’s exceedingly low oral bioavailability, therefore, genistein and daidzein were selected for subsequent molecular docking analysis. The resultant receptor-ligand complexes formed interactions primarily via van der Waals forces, Pi-Pi stacking, and Pi-alkyl bonds (Fig. 6).

**Table 2:** Binding energy.

	Weighted binding energy		
	Level 1	Level 2	Level 3
Daidzein	-8.525	-8.525	-8.408
Genistein	-8.3	-8.3	-8.18
Apigenin	-8.35	-8.35	-8.236
Glycitein	-8.275	-8.275	-8.164
Biochanin A	-8.225	-8.225	-8.108

The Weighted Binding Energy was calculated as the linear combination of the individual binding energies, scaled by their respective biological expression ratios: Weighted Binding Energy = [Binding Energy (AKT1) × w1] + [Binding Energy (SRC) × w2]. The weights w1 and w2 correspond to the normalized relative expression levels of AKT1 and SRC, which were sourced from the NCBI Gene Expression portal (Table S2). Level 1 and 2 (lung 10 and 17 wk): w1 = 0.75 and w2 = 0.25; Level 3 (lung 20 wk): w1 = 0.72 and w2 = 0.28, w1 + w2 = 1, wk was weeks of gestational time.

### Anti-inflammatory and antioxidant efficacy of genistein and daidzein

Based on the network pharmacology screening and molecular docking results, genistein and daidzein were selected as the primary candidate bioactive constituents in SSP for subsequent pharmacological validation against influenza. Their protective effects were assessed on the A549 cell line model of influenza-associated respiratory inflammation and oxidative stress. Concentration screening determined the working concentration of these constituents. For the anti-inflammatory model (LPS-induced): 1.562 µM for genistein and 1.752 µM for daidzein; for the antioxidant model (H₂O₂-induced): 1.577 µM for genistein and 2.054 µM for daidzein.

**Fig. 7:**
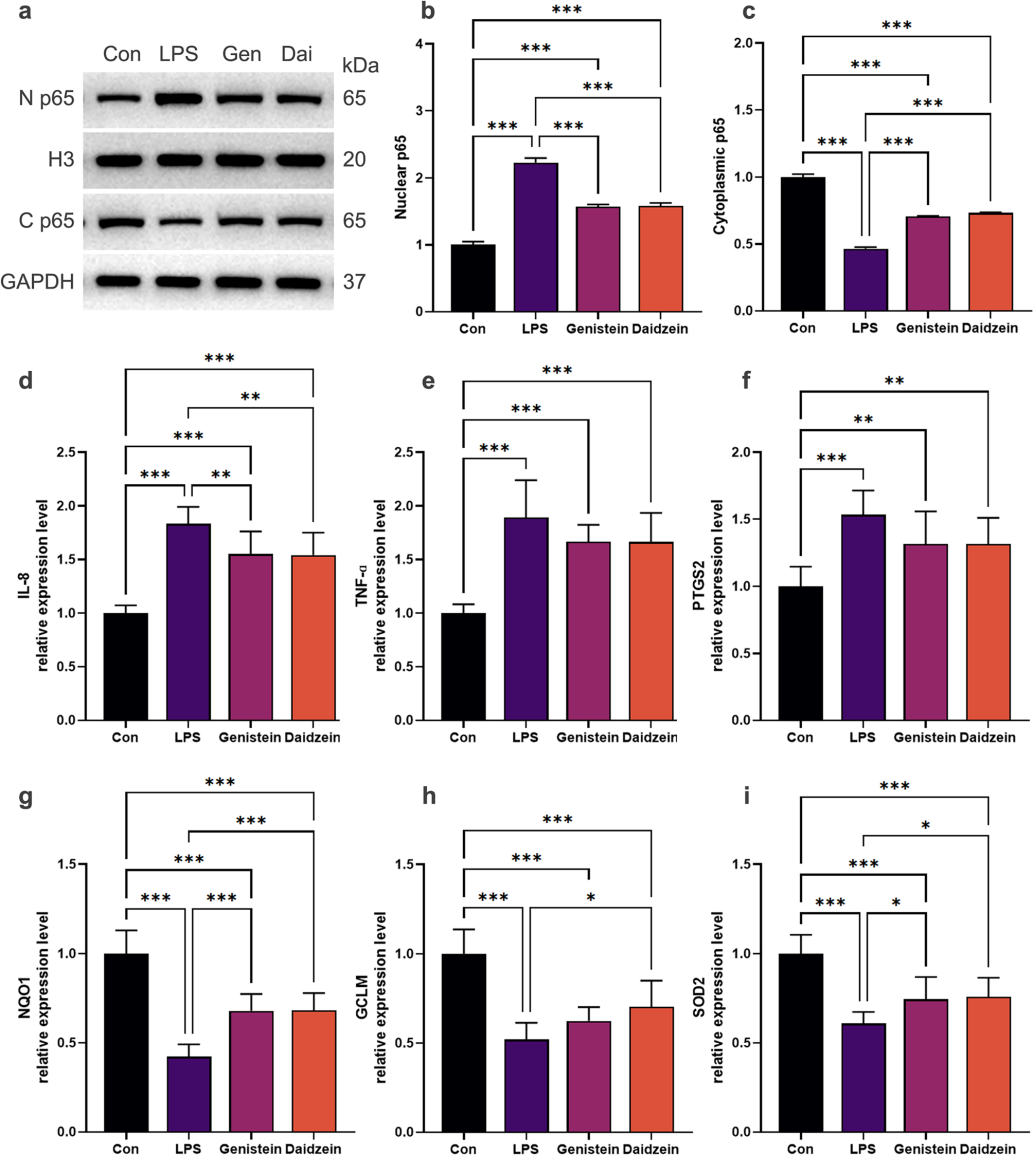
Anti-inflammatory and antioxidant efficacy of genistein and daidzein. (**a**–**c**) Western blot analysis of NF-κB p65 in nuclear and cytoplasmic fractions following H_2_O_2−_ or LPS-induced treatment with or without genistein/daidzein pretreatment. (**d**–**i**) qRT-PCR analysis of pro-inflammatory (IL-8, TNF-α, PTGS2) and antioxidative (NQO1, GCLM, SOD2) mediators mRNA expression. Genistein and daidzein markedly regulated the nuclear-translocation and transcription of these mediators in both H_2_O_2−_ and LPS-induced models. The data were represented as the mean ± SD (*n* = 3), ANOVA/Tukey’s-test. * *p* < 0.05, ** *p* < 0.01, *** *p* < 0.001.

Genistein and daidzein markedly suppressed the nuclear translocation of NF-κB p65 (Fig. 7a–c) and downregulated the mRNA expression of pro-inflammatory mediators, including IL-8, TNF-α, and PTGS2 (Fig. 7d–f). Concurrently, the transcription of key antioxidant regulatory genes was significantly upregulated, including NQO1, GCLM, and SOD2 (Fig. 7g–i).

**Fig. 8:**
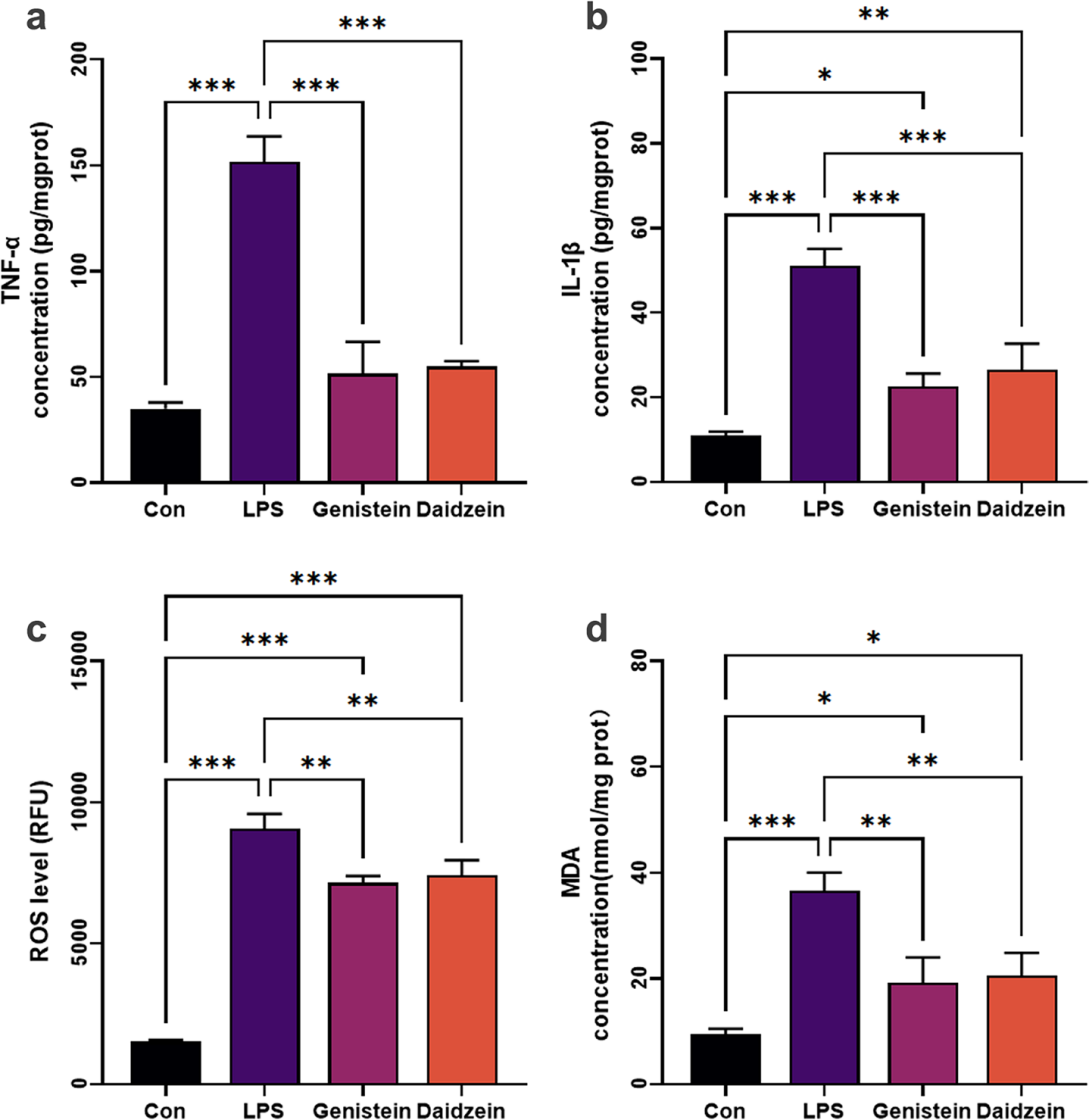
Genistein and daidzein inhibited inflammatory and oxidant effectors expression. (**a**, **b**) ELISA quantification of extracellular TNF-α and IL-1β secretion. (**c**, **d**) biochemical assay results for oxidant ROS and MDA level. The data were represented as the mean ± SD (*n* = 3), ANOVA/Tukey’s-test. * *p* < 0.05, ** *p* < 0.01, *** *p* < 0.001.

Consistent with these molecular changes, extracellular secretion of TNF-α and IL-1β (Fig. 8a,b) and intracellular levels of ROS and MDA were substantially decreased (Fig. 8c,d) in both models following treatment with genistein and daidzein. These findings collectively indicate that genistein and daidzein exert protective effects in A549 cells against influenza-like oxidative stress and inflammation through inhibition of NF-κB signaling, suppression of pro-inflammatory cytokine production, and enhancement of endogenous antioxidant defenses.

### Quantitative analysis and HPLC fingerprint of SSP samples

Network pharmacology and molecular docking analysis identified genistein and daidzein as key active constituents, which were subsequently utilized to evaluate the SSP products. The total content of genistein and daidzein exceeded 0.040% in the majority of samples. Additionally, routine quality assessments, encompassing impurity, moisture, ash, and extractum content, fully complied with the standards stipulated in the Chinese Pharmacopoeia (Tables 3 and 4).

**Table 3:** Daidzein and genistein content in SSP.

No.	Constituents	Content (%)	Total content (%)
1	Daidzein	0.073	0.107
	Genistein	0.034	
2	Daidzein	0.032	0.052
	Genistein	0.020	
3	Daidzein	0.067	0.104
	Genistein	0.037	
4	Daidzein	0.076	0.119
	Genistein	0.043	
5	Daidzein	0.083	0.126
	Genistein	0.043	
6	Daidzein	0.031	0.051
	Genistein	0.020	
7	Daidzein	0.026	0.043
	Genistein	0.017	
8	Daidzein	0.024	0.043
	Genistein	0.019	
9	Daidzein	0.018	0.032
	Genistein	0.014	

The total content was a sum of daidzein and genistein results.

**Table 4:** Routine quality assessment results.

No.	Sample (g)	Impurity (%)	Moisture (%)	Ash (%)	Extractum (%)
1–1	83.566	3.0	8.6	5.0	15.7
1–2	76.457	2.4	8.7	5.0	15.6
2 − 1	84.157	2.9	9.5	5.7	15.5
2–2	58.198	2.1	9.4	5.6	15.5
3 − 1	68.144	2.0	8.9	4.0	15.6
3 − 2	63.398	1.9	8.9	3.9	15.7
4 − 1	56.558	1.9	7.5	4.9	16.4
4 − 2	55.971	1.8	7.7	4.9	16.4
5 − 1	88.232	2.8	8.3	7.0	15.7
5 − 2	71.128	2.5	8.4	7.0	15.7
6 − 1	74.434	1.5	8.8	6.8	15.5
6 − 2	90.907	1.7	8.8	6.8	15.6
7 − 1	86.48	1.9	7.9	6.9	15.9
7 − 2	81.736	1.6	8.1	6.9	15.9
8 − 1	76.893	2.4	9.7	5.2	15.4
8 − 2	73.139	2.8	9.7	5.2	15.4
9 − 1	91.713	2.2	9.4	6.5	15.4
9 − 2	82.956	2.4	9.5	6.5	15.5

**Fig. 9:**
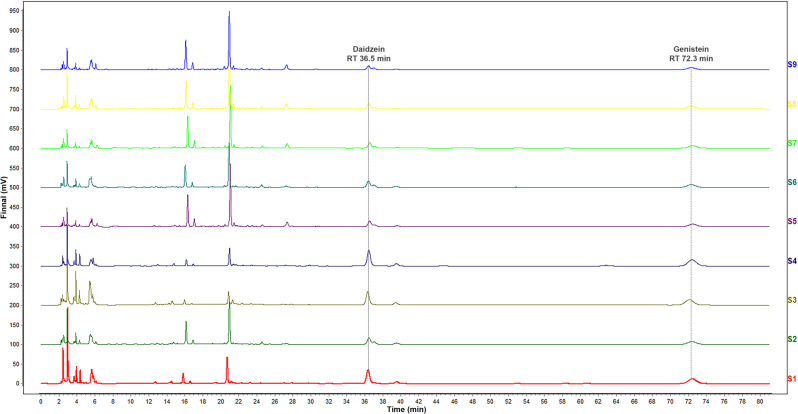
HPLC fingerprint chromatograms of different batches of SSP samples. Sample 1 (bottommost trace) served as the reference chromatogram, with genistein and daidzein identified as the two shared characteristic peaks.

Similarity evaluation of the chromatograms from nine batches of SSP samples was conducted via multi-point calibration and marker peak matching. Two common peaks were identified: genistein and daidzein (Fig. 9). The similarities to the reference spectrum were 0.995, 0.999, 1.000, 1.000, 0.999, 0.999, 0.998, 0.990, and 0.989, respectively, indicated stable quality across SSP batches produced under varying fermentation processes.

To determine the optimal fermentation parameters, preliminary single-factor experiments were conducted to assess the effects of water-to-material ratio and temperature. When the water-to-material ratio exceeded 20:1 (v/w), excessive decoction liquid caused the soybeans to remain overly immersed, which hindered proper microbial activity. Similarly, fermentation temperatures above 30 °C (e.g., 40–50 °C tested in preliminary experiments) led to undesirable formation of yellow or white coatings accompanied by strong putrid odors, ultimately resulting in fermentation failure.

Based on these preliminary findings, the fermentation conditions were further optimized and validated through HPLC quantification and fingerprint chromatogram similarity analysis. Through HPLC quantification and fingerprint chromatogram similarity analysis, the optimal fermentation conditions for SSP were established. During co-decoction of adjuncts with black soybeans, a water-to-material ratio of 20:1 (v/w) was applied, with fermentation maintained at (30 ± 2) °C under open conditions, yielding the highest contents of genistein and daidzein (Table 3, Batch 5). Under these parameters, the contents of daidzein, genistein, and their total were maximized. The resulting SSP exhibited an elliptical, slightly flattened shape, with a dark black epidermis featuring uneven wrinkles, a brownish-black cross-section, a pronounced aromatic fragrance (Fig. 1c), and a mildly sweet taste. Compared to traditional SSP fermentation processes, the optimized protocol significantly shortened the fermentation cycle while markedly enhancing genistein and daidzein contents. Furthermore, experiments by fellow group members demonstrated that the optimized SSP outperformed commercially available products in content determination (Table S4).

## Discussion

Semen Sojae Praeparatum (SSP), a fermented black soybean, has historically been valued for its ability to dispel exterior pathogens, clear heat, resolve toxins, and alleviate irritability, aligning with its application in treating “Wen Bing”, which exhibits symptoms parallel to those of influenza^23^. Numerous TCM formulations incorporating SSP, such as Cong Chi Tang, Yin Qiao San and Wei Rui Tang, have been utilized for influenza management^24–28^. These formulations leverage SSP’s properties to address cytokine storms and secondary complications, offering multi-target coverage against variant pathogens. However, the current fermentation processes of SSP often suffer from inconsistencies in microbial control and parameter optimizatio^29^, leading to variable bioactive profiles; thus, selectively employing anti-influenza constituents as evaluative markers to refine SSP fermentation protocols emerges as a pivotal strategy for ensuring standardized efficacy.

Network pharmacology identified genistein and daidzein as key therapeutic constituents against influenza. These constituents intersect with multiple targets, highlighting hub proteins such as TNF, AKT1, EGFR, SRC, and MMP9 in pathological domains including cytokine storm, viral replication, pulmonary remodeling, and endothelial dysfunction. Influenza infection triggers the secretion of TNF-α, which binds to TNFR1/2^30^, activating the PI3K/AKT pathway and leading to the phosphorylation of AKT1 at Thr308/Ser473^31,32^, thereby regulating apoptosis. This pathological process transactivates EGFR via ligand shedding, recruiting SRC to facilitate dimerization^33–35^, amplifying cytokine production via NF-κB, and escalating inflammation^36^. The TNF-AKT1-EGFR-SRC axis upregulates MMP9 via NF-κB, cleaving the extracellular matrix (ECM), disrupting alveolar structures, promoting immune infiltration, fibrosis, and secondary infections, and ultimately culminating in influenza-derived acute respiratory distress syndrome (ARDS) and systemic complications^37–40^. The bioactive constituents genistein and daidzein exhibited strong binding affinities to influenza-related targets-particularly AKT1 and SRC, which are highly expressed in the lungs and critical for influenza pathogenesis in susceptible populations-mediated by van der Waals forces, Pi-Pi stacking, and Pi-alkyl interactions. These interactions disrupted downstream signaling pathways, significantly inhibiting the nuclear translocation of NF-κB p65, suppressing the transcription (IL-8, TNF-α and PTGS2) and expression of pro-inflammatory cytokines (TNF-α and IL-1β), upregulating the transcription of key antioxidant defense genes (NQO1, GCLM and SOD2), and attenuating the generation of oxidative stress products (ROS and MDA). Collectively, these effects suggest that genistein and daidzein may protect pulmonary epithelial cells during influenza-induced pulmonary infection, thereby highlighting SSP’s potential antiviral and anti-inflammatory activity against influenza.

Guided by these findings, genistein and daidzein were selected as indicator constituents for subsequent fermentation optimization, owing to their high binding affinities and favorable oral bioavailability. Prior to conducting the formal optimization experiments, preliminary single-factor experiments revealed that high water-to-material ratios exceeding 20:1 led to excessive immersion of the soybeans, violating the traditional Chinese medicine processing principle of “Yao Tou Zhi Jin” (complete permeation of medicinal material with full absorption of liquid accessories) and thereby normal microbial activity and fermentation. Similarly, fermentation temperatures above 30 °C resulted in undesirable microbial growth accompanied by strong putrid odors, ultimately leading to fermentation failure. The quality of the nine batches of fermented SSP, prepared according to a systematic factorial design combining the key process parameters (water-to-material ratio, temperature, and sealing condition), was evaluated by HPLC fingerprinting. All batches exhibited high chromatographic similarity and total genistein and daidzein contents that exceeded the requirements of the Chinese Pharmacopoeia. Consequently, the optimal fermentation parameters incorporated a water-to-material ratio of 20:1, a temperature of (30 ± 2) °C, and open conditions. Samples produced under these optimized fermentation parameters exhibited higher contents of the indicator constituents (genistein and daidzein) compared to commercial products. The methodologies and parameters established in this study demonstrate strong process controllability and commercial applicability, providing a valuable reference for the future industrial production of fermented soybeans.

## Conclusion

This study elucidated the anti-influenza potential of SSP by identifying genistein and daidzein as pivotal constituents that target critical pathological hubs, particularly AKT1 and SRC, key lung-expressed mediators in vulnerable populations. Concurrently, these interactions disrupted downstream signaling pathways and observed effects on NF-κB p65 nuclear translocation, thereby highlighting SSP’s potential anti-inflammatory and antioxidant activity against influenza. HPLC fingerprinting confirmed chemical consistency across optimized fermentation batches. The refined protocol (water-to-material of 20:1, temperature of 30 ± 2 °C, and open fermentation) significantly enhanced bioactive content and outperformed commercial samples. These findings reinforce SSP’s therapeutic value in Traditional Chinese Medicine-based influenza management while providing a scientifically robust, industrially viable standardization strategy, thereby facilitating its broader clinical integration and large-scale production.

## Supplementary Information

Below is the link to the electronic supplementary material.

Supplementary Material 1

## Data Availability

All data generated or analyzed during this study are available. It shall be provided by the corresponding author on reasonable request.
