# Supplementary material for: Network pharmacology-guided optimization of Semen Sojae Praeparatum fermentation for enhanced anti-influenza efficacy
Source: Sci Rep. 2026 Apr 9;16:16726. doi: 10.1038/s41598-026-47737-7 (PMC13223287; doi:10.1038/s41598-026-47737-7)
Supplement: Supplementary file 1 — Supplementary Material 1 [file 41598_2026_47737_MOESM1_ESM.docx]

**Supplementary Material**

**Supplementary Figures and Tables**

**
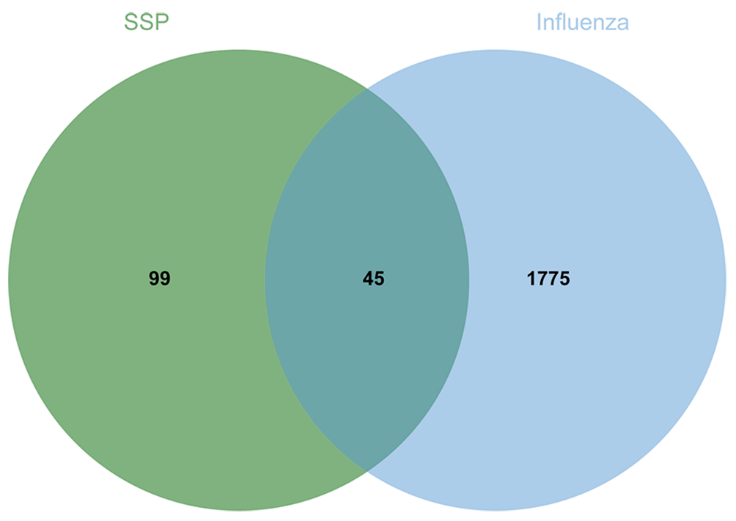
**

**Supplementary Figure 1.** Venn Diagram of SSP components and anti-influenza target. The number of chemical constituents’ target of SSP and anti-influenza target was 144 and 1820, the intersection of them was 45.

**
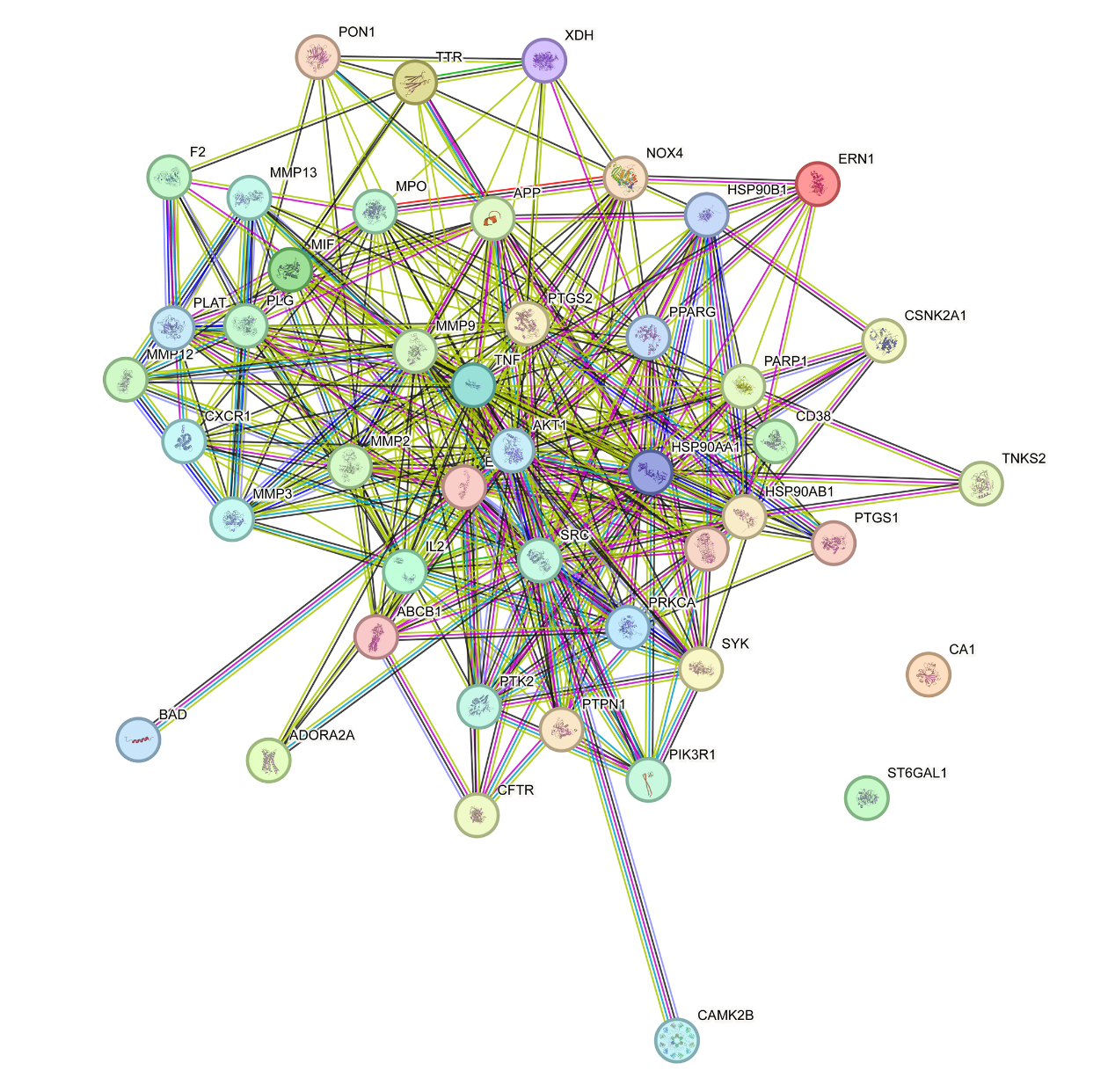
**

**Supplementary Figure 2.** PPI network of potential anti-influenza targets in SSP.

**Supplementary Table 1. qPCR primer sequence**

| Gene | 5’-3’ | |  |
| --- | --- | --- | --- |
| HMOX1-F | | GCAGGAGGTCATCCCCTACACAC | |
| HMOX1-R | | TCTGGGCAATCTTTTTGAGCACC | |
| NQO1-F | | GGTGGTGGAGTCGGACCTCTATG | |
| NQO1-R | | GCCTTTCAGAATGGCAGGGACT | |
| GCLM-F | | GCTGTGTGATGCCACCAGATTTG | |
| GCLM-R | | ATGACCGAATACCGCAGTAGCCA | |
| SOD2-F | | TCTGGACAAACCTCAGCCCTAAC | |
| SOD2-R | | GAAACCAAGCCAACCCCAAC | |
| IL-6-F | | CCTTCGGTCCAGTTGCCTTCT | |
| IL-6-R | | TTCTGCCAGTGCCTCTTTGCT | |
| IL-8-F | | AAGACATACTCCAAACCTTTCCACC | |
| IL-8-R | | CTTCAAAAACTTCTCCACAACCCTC | |
| Tnf-α-F | | CCAGGGACCTCTCTCTAATCAGC | |
| Tnf-α-R | | AGGCTTGTCACTCGGGGTTC | |
| IL-1B-F | | TGAAATGATGGCTTATTACAGTGGC | |
| IL-1B-R | | TGTAGTGGTGGTCGGAGATTCGTAG | |
| PTGS2-F | | AAACCGAGGTGTATGTATGAGTGTG | |
| PTGS2-R | | ATCCCTTGAAGTGGGTAAGTATGTAG | |
| β-actin-F | | TGGCACCCAGCACAATGAA | |
| β-actin-R | | GAAGCATTTGCGGTGGACG | |

**Supplementary Table 2. Anti**-**influenza compounds of SSP**

| No. | Compound Name | Molecular Weight (Da) | H-Bond Donors | H-Bond Acceptors | LogP |
| --- | --- | --- | --- | --- | --- |
| 1 | Syringaldehyde | 182.17 | 1 | 4 | 0 |
| 2 | Syringic Acid | 198.17 | 2 | 5 | 1 |
| 3 | 2,3,5,6-Tetramethylpyrazine | 136.19 | 0 | 2 | 1.3 |
| 4 | Isoflavone | 222.24 | 0 | 2 | 3.2 |
| 5 | Daidzin | 416.40 | 5 | 9 | 0.7 |
| 6 | Glycitin | 446.40 | 5 | 10 | 0.6 |
| 7 | Biochanin A | 284.26 | 2 | 5 | 3.0 |
| 8 | Apigenin | 270.24 | 3 | 5 | 1.7 |
| 9 | Genistein | 270.24 | 3 | 5 | 2.7 |
| 10 | Daidzein | 254.24 | 2 | 4 | 2.5 |
| 11 | Glycitein | 284.26 | 2 | 5 | 2.4 |

**Supplementary Table 3. Target gene expression level of influenza in lung**

|  | Expression level | | |
| --- | --- | --- | --- |
|  | lung 10 wk | lung 17 wk | lung 20 wk |
| AKT1 | 5.27 | 7.44 | 5.41 |
| TNF | 0.0216 | 0.0679 | 0.0225 |
| ESR1 | 0.128 | 0.0537 | 0.0538 |
| SRC | 1.95 | 2.73 | 2.05 |
| MMP9 | 0.0162 | 0.0742 | 0.0308 |

The data were obtained from https://www.ncbi.nlm.nih.gov/gene/

**Supplementary Table 4. Total content of daidzein and genistein**

| Sample No. | Genistein + Daidzein (%) | Total Content (m/m%) |
| --- | --- | --- |
| 1 | 0.026 | 0.090 |
| 2 | 0.014 | 0.104 |
| 3 | 0.012 | 0.094 |
| 4 | 0.107 | 0.159 |
| 5 | 0.052 | 0.142 |
| 6 | 0.104 | 0.132 |
| 7 | 0.119 | 0.152 |
| 8 | 0.126 | 0.144 |
| 9 | 0.051 | 0.141 |
| 10 | 0.043 | 0.158 |
| 11 | 0.043 | 0.152 |

No.1-3 samples were commercial products and No.4-11 samples were prepared in lab.
